# Cardiac Electrophysiology

**DOI:** 10.1155/2013/909746

**Published:** 2013-08-20

**Authors:** Yanggan Wang, Yimei Du, Xun Ai

**Affiliations:** ^1^Department of Cardiology, Zhongnan Hospital, Wuhan University, Wuhan 430071, China; ^2^Department of Cardiology, Xiehe Hospital of Huazhong University of Science and Technology, Wuhan 430022, China; ^3^Department of Physiology, Loyola University Chicago, Maywood, IL 60153, USA

Recently, significant achievements have been made in cardiac electrophysiology research, including Ca^2+^ regulatory pathways, membrane ion channel-related intracellular signaling, and electrical remodeling in cardiomyopathies. The rapid progress in the basic research has led to applications of numerous new methods and strategies to clinical diagnoses and treatments. The efficacy of antiarrhythmic pharmacotherapy remains a major challenge, but recent insights into complex mechanisms of electrical remodeling and new therapeutic strategies have raised the prospect of targeting arrhythmogenic substrates.

This special issue introduces recent progress in this area with special focus on atrial fibrillation, one of the most common arrhythmias in structural heart diseases. Papers published in this issue include basic research in ion channels, polymorphisms associated with lone atrial fibrillation, low-level autonomic stimulation on prevention of atrial fibrillation, new clinical methods for diagnosis and treatment of atrial fibrillation, and the pivotal role of cardiac imaging and electrocardiogram in assessing the effectiveness of cardiac resynchronization therapy. Due to volume limits, several important areas have not been included in this issue. However, we plan to discuss these topics in future issues. 

*Yanggan  Wang* *Yanggan  Wang* *Yimei  Du* *Yimei  Du* *Xun  Ai* *Xun  Ai*

